# Influence of season and social context on male giant panda (*Ailuropoda melanoleuca*) vocal behaviour

**DOI:** 10.1371/journal.pone.0225772

**Published:** 2019-11-26

**Authors:** Benjamin D. Charlton, Megan A. Owen, Xiaoping Zhou, Hemin Zhang, Ronald R. Swaisgood

**Affiliations:** 1 Institute for Conservation Research, San Diego Zoo Global, San Diego, California, United States of America; 2 China Conservation and Research Center for the Giant Panda, Wolong, Sichuan, China; Institute of Animal Science, CZECH REPUBLIC

## Abstract

Documenting the different social and behavioural contexts that vocalisations are produced in remains an important step towards understanding the functional relevance of specific call types in a given species’ vocal repertoire. In this study we investigated whether seasonal differences and the presence or absence of male and female conspecifics influence the production of male giant panda vocal signals. To this end, captive male giant pandas were observed during and outside of the breeding season in three social contexts: only male conspecific neighbours, only female conspecific neighbours, and a context with no neighbours. We found that males were more likely to bleat, chirp, honk and moan during the breeding season, and showed a tendency to growl more outside of the reproductive period. The contextual analysis revealed that bleats were more likely to be produced by males when opposite-sexed conspecifics are in close attendance during the breeding season. Conversely, males were more likely to chirp when neighboured by males than females or no neighbours. In addition, males were more likely to honk in the absence of neighbouring conspecifics during the breeding season, raising the possibility that these calls function to signal location and gain the attention of potential mates. Moans were produced more often when male giant pandas had male than female neighbours during the breeding season, which may reflect mild aggression towards these same-sexed rivals, whereas the production of barks and growls did not vary according to season or the sex of conspecific neighbours. Our findings underscore the importance of male giant panda bleats for coordinating reproduction and promoting contact with potential mating partners in this non-gregarious species, and yield fresh insights into the function of male honks that warrant further investigation. They also provide a basis for comparison with free-ranging giant panda vocal behaviour that could potentially inform conservation efforts.

## Introduction

A number of studies highlight how mammal vocal signals contain information about the caller’s physical attributes [1–5] and physiological state [6–8], and whether receivers attend to this acoustic variation in a range of social and sexual contexts [9]. In species that produce a range of vocalisations it is also important to document how the production of different call types varies according to the contexts of emission [10–16]. For instance, exclusive or predominant use of a specific call type during the breeding season is consistent with a sexual role [17, 18], whereas loud calling when conspecifics are not within close range suggests a long range function, such as alerting social group members to the presence of predators [19], maintaining contact with widely spaced social group members [20, 21] advertising location [16, 22], and/or attracting potential mating partners [23, 24]. In this study we examined male giant panda (*Ailuropoda melanoleuca*) vocal behaviour during and outside of the breeding season to document context-related differences in call production, and in doing so, further knowledge about the function of discrete call types in this conservation-reliant species.

Giant pandas (*Ailuropoda melanoleuca*) are asocial bamboo foragers that typically avoid other adult conspecifics [25–27]. The giant panda’s solitary nature [25–27] means that effective communication is vital for locating potential mates and for coordinating reproduction [25, 28]. Research to date indicates that chemical signals allow giant pandas to determine the relatedness [29], identity, [30] sex [31], reproductive state [32] and competitive status of signallers [33, 34], prior to face-to-face encounters. Once close range contact between potential mates has been established, the giant panda’s diverse vocal repertoire [35, 36] is likely to attain more importance for signalling short-term fluctuations in arousal levels and behavioural intention in the lead up to copulation [37].

The most conspicuous giant panda vocalisation is a frequency-modulated bleat (S1 Audio) that appears to promote contact between individuals in the lead up to copulation [38–40]. A recent series of studies has also revealed that giant panda bleats contain information on the caller’s identity [41], body size, age, and sex [42], hormonal quality [43], and motivational state [44], and demonstrated that male and female giant pandas attend to this potentially important information during the breeding season [45–47]. Oestrous female giant pandas also produce high-pitched chirp vocalisations [38, 48, 49] (S2 Audio) that allow males to target mating attempts on these individuals, and maximise the chances of conception [50]. In addition, male giant pandas also chirp [36, 39], albeit very rarely, and both sexes moan (S3 Audio), bark (S4 Audio), and growl (S5 Audio) during close-range interactions, and produce honks (S6 Audio) with and without conspecifics in close attendance [35, 36, 39]. It is generally assumed that barks, growls and roars are aggressive calls that are produced during agonistic encounters, squeals are submissive calls produced during and after an aggressive interaction, and moans denote a mildly aggressive or ambivalent mood [39]. Giant panda honks are thought to signal frustration when an animal is prevented from achieving a desired goal, both social and non-social [39].

Early studies of giant panda vocal signal usage were based primarily on a breeding pair at National Zoological Park, Washington [38, 39, 48]. While this pioneering work provided key insights into the potential function of different call types, the ability to generalise the results is restricted by the sample size of only two animals, and a lack of observational data on interactions between individuals of the same sex. More recent work using a larger sample of giant pandas has shown that females vary the production of bleats, but not chirps or moans, according to the presence or absence of neighbouring conspecifics of each sex [51]. It is not known whether male giant pandas vary the production of different call types according to social context. In addition, male giant pandas experience a rise in testosterone levels during the breeding season [52, 53], particularly when they encounter potential mates [53], and this may be expected to increase the production of call types most relevant to sexual behaviour and reproduction. Despite this, the affect of season on male giant panda vocal behaviour has not been examined.

To address this knowledge gap we used longitudinal data collected over a seven-year period to determine whether seasonal differences and variation in the social context of emission influence the production of male giant panda vocal signals. We predicted that males would produce significantly more bleats and chirps during the breeding season than outside of this time. We had no *a priori* seasonal predictions for the more aggressive call types in the giant panda’s repertoire (moans, honks, barks, growls and roars). Our second aim was to determine whether the presence or absence of male and female conspecifics influences the production of different male vocalisations. We predicted that male giant pandas housed next to females during the breeding season would produce more bleat and chirp vocalisations to promote contact and signal friendly intent to potential mating partners. We also predicted that if honks are used to signal location in reproductive contexts they will be produced more often when male giant pandas are without neighbours during the breeding season when contact with potential mates is sought. In contrast, males housed next to male conspecifics were expected to produce more aggressive vocalisations, such as moans, barks, growls and roars.

## Materials & methods

### Ethical statement

This study followed the ASAB/ABS guidelines for the use of animals in research, and was approved by the San Diego Zoo IACUC. None of the procedures used affected the housing, diet or management of the animals.

### Subjects and study site

The data for this study were collected over a seven-year period from 1996 to 2003 at the China Research and Conservation Centre for the Giant Panda (CRCCGP) in the Wolong National Nature Reserve, Sichuan, China. Breeding season observations were conducted during February-May. This is the time when females typically come into oestrus and is recognized as the giant panda’s reproductive period [25, 54]. Non-breeding season observations were conducted during August-December, and hence, at least one month outside of the breeding season. We included 17 adult male giant pandas (aged 4–21 years, mean age = 10.7 years) (Table 1) and 33 neighbouring animals in the study (23 females, 10 males). None of the female neighbours were displaying behavioural signs of oestrus during the breeding season observations.

**Table 1:** The number of 45-minute observation sessions for each subject in the three neighbour contexts. Entering subject identity and observation year as random factors in the GLMMs allowed us to control for the uneven subject participation across neighbour contexts.

	Neighbour context		
Subject	Female	Male	None
An An	0	0	9
Da Di	151	0	9
Di Di	121	13	12
Gao Gao	43	0	2
Gu Gu	0	0	4
Lin Lin	59	0	6
Lin Nan	15	6	6
Long Long	3	1	0
Lu Lu	0	4	5
Pan Pan	119	11	4
Peng Peng	1	5	4
Ping Ping	10	0	0
Qing Qing	0	3	8
Shi Shi	0	0	9
Xi Meng	117	0	10
Xin Xing	155	0	4
Zhuang Zhuang	31	0	33
**Total**	**823**	**43**	**125**

The giant pandas at the CRCCGP were fed a diet of bamboo, bread, and milk, and provided with water *ad libitum*. The study population consisted of both wild-caught and captive-born animals that were located in separate enclosures. Each of the enclosures had an outside yard measuring approximately 10*10 and an indoor 3*5 m den area. Five animals were kept in enclosures less than half this size and two had simultaneous access to a large outdoor yard approximately 1 ha in size. The animals had access to one or two neighbours housed in adjoining enclosures through heavy gauge wire mesh and bars that offered good visual and vocal access to neighbours, whilst precluding all but minimal direct physical contact.

### Collection of behavioural data and definition of social contexts

We conducted 993 focal observation sessions that each lasted for 45 minutes during (N = 820) and outside (N = 173) of the breeding season. Care was taken to avoid conducting observations during periods of construction and/or times when other disturbances were likely (due to keeper interferences or general husbandry, for example). Focal sessions were conducted between 0645 and 1000 hours and 1430 and 1600 hours, which are the times when the giant pandas at the CRCCGP are typically most active. Vocal behaviour was recorded using 1–0 sampling at one-minute intervals [55, 56]. The end of each one-minute sample interval was denoted by an audible signal from a watch. An observer recorded the occurrence and type of male vocal behaviour during each interval. The score obtained reflects the number of intervals during an observation session wherein focal animals produced different call types across the 45-minute observation periods. Because squealing was only observed once and no roars were observed during the study period these call types were not included in the subsequent analyses. The social context in the current study was the gender of neighbouring animals and constituted three groups: male giant pandas with male neighbours in adjoining enclosures, male giant pandas with female neighbours in adjoining enclosures, or male giant pandas with no neighbouring animals in adjoining enclosures. All observers were trained to recognise the different vocalisations using an ethogram (Table 2) and required to pass reliability tests (Cohen’s kappa > 0.85 agreement) [55].

**Table 2:** Ethogram of male giant panda vocal behaviour. Exemplars of the different call types are provided as supplementary material. Refer to methods section for details about the 1–0 sampling protocol.

Vocalisation	Description
Bleat	A twittering, goat-like call of variable length (1-3s).
Chirp	Short, tonal (free of distortion), high-pitched call rising and descending in pitch
Moan	Low-pitched, low-to-medium amplitude, call of variable duration. Often has several short starting elements. “Ranges from a soft hoot and softly repeated bu-bu-bu to a low-pitched moo, whiny groan and long drawn-out moan rising and falling in pitch” (Schaller et al, 1985).
Honk	Short (< 0.5s), tonal, low-pitched, nasal call, falling in pitch. Almost always produced repetitively in a series, generally lasting for several minutes.
Bark	Short, (0.1–0.3s), fairly noisy, similar to dog bark.
Growl	Long, noisy, low-pitched call similar to a dog growl

### Statistics

To examine the data generalized linear mixed models (GLMMs) were computed using R v3.2.3 (R Foundation for Statistical Computing, Vienna, Austria). The GLMMS were fitted with a Poisson distribution and logistic link function. A Poisson distribution is the most appropriate for dependent variables that represent a count of occurrences over time [57]. For the GLMM analysis we entered the occurrence data for each of the different male vocalisations observed across the study (bleat, chirp, moan, honk, bark and growl) as dependent variables, and season (breeding or nonbreeding) and neighbour context (male, female, none) entered as categorical predictor variables. In addition, the identity of the focal animal, the year the research was conducted, and the researcher that conducted the observations were entered as random factors in the GLMMs. Random factors represent a random sample of a larger set of potential events, (e.g. the subjects in our experiment) and are distinguished by the fact that we are only interested in the variance they explain and not their actual parameter values [58]. By entering the identity of the focal animal as a random factor we therefore control for the uneven subject participation across conditions, avoid any problems with temporal pseudoreplication (repeated measures taken from the same individual), and make the unit for statistical inference the number of observations, rather than the number of individuals [50, 59, 60]. To avoid over-fitting, the interaction term season*neighbour context was only retained in a given GLMM when it was statistically significant. Post-hoc pairwise comparisons using Tukey's HSD were used to evaluate significant differences between the neighbour contexts. Significance levels were set at *P* < 0.05.

## Results

### Bleats

**Fig 1:**
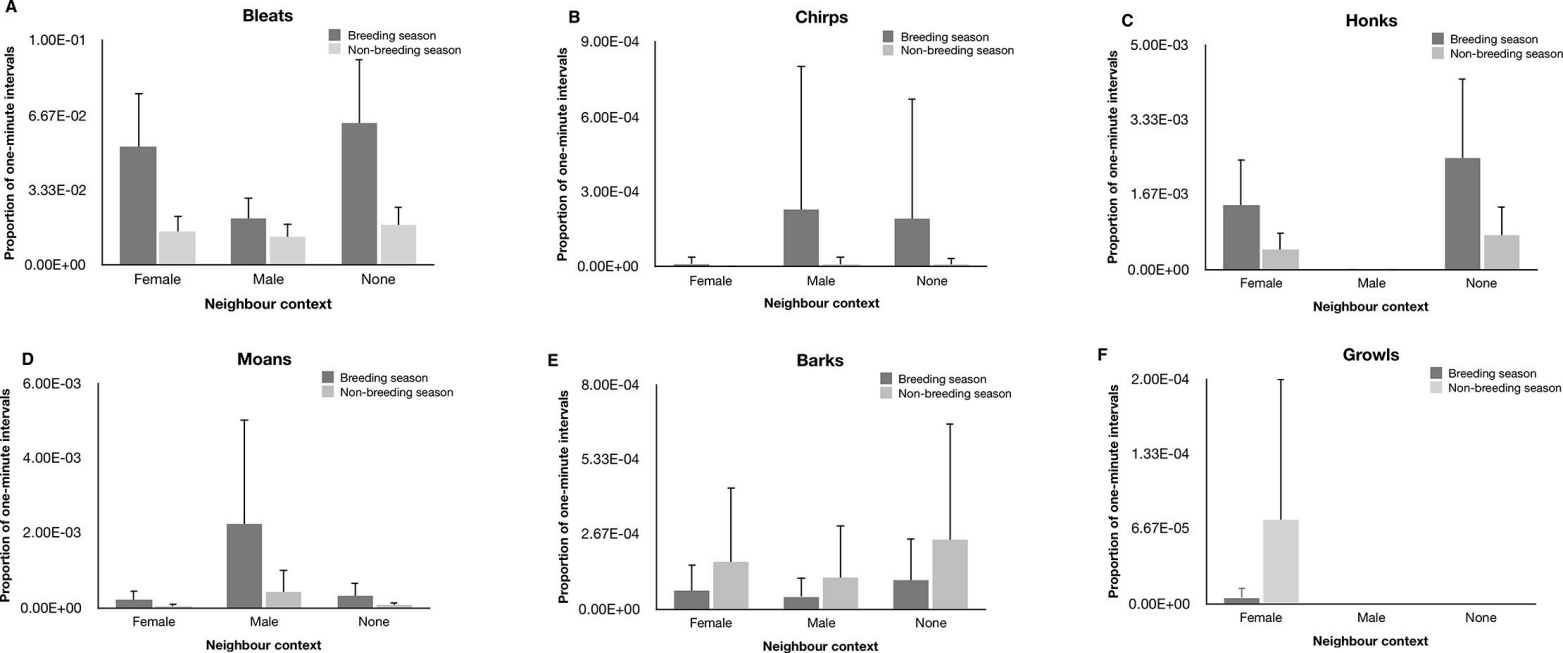
Error bar charts showing the effect of neighbour context on male vocal behaviour during and outside of the breeding season. Back-transformed estimated marginal means ± SE of the proportion of intervals in which males were (a) bleating, (b) chirping, (c) moaning (d) honking (e) barking (f) and growling during the 45 minute observation sessions are presented [55].

We found that male giant pandas were more likely to bleat during the breeding season (Wald χ^2^_1, 992_ = 169.4, *P* < 0.001). The sex of neighbouring conspecifics also had a significant effect on the production of male bleats (Wald χ^2^_2, 991_ = 27.6, *P* < 0.001) (Fig 1A). Post hoc analyses revealed that males were significantly more likely to bleat when they were housed next to females than males (*P* < 0.001). In addition, males without neighbours were more likely to bleat than those that were housed next to other males (*P* < 0.001) (Fig 1A). A statistically significant difference in bleating was not revealed for males housed next to females versus no neighbours (*P* = 0.059) (Fig 1A). The interaction term season*neighbour context was significant (Wald χ^2^_2, 991_ = 12.1, *P* = 0.002). Examination of the estimated marginal means reveals that the pattern of increased male bleating when housed next to females and no neighbours was most evident during the breeding season (Fig 1A).

### Chirps

Males chirped more during the breeding season than the non-breeding season (Wald χ^2^_1, 992_ = 7.8, *P* = 0.005). Neighbour context was also a significant predictor of male chirping behaviour (Wald χ^2^_2, 991_ = 36.5, *P* < 0.001). The post-hoc analyses indicated that males without neighbours were more likely to chirp than those neighboured by females (*P* < 0.001) (Fig 1B). In addition, males with male neighbours chirped more than those with female neighbours (*P* = 0.019). No difference in chirping behaviour was detected for males housed next to males versus no neighbours (*P* = 0.982) (Fig 1B).

### Honks

Male giant pandas were more likely to honk during than outside of the breeding season (Wald χ^2^_1, 992_ = 5.3, *P* = 0.021). In addition, the occurrence of honking varied across neighbour contexts (Wald χ^2^_2, 991_ = 9.1, *P* = 0.011), with males significantly more likely to produce honks when they had no neighbours than when they had female or male neighbours (both *P* < 0.001) (Fig 1C). Males also honked more when housed next to female neighbours versus male neighbours (*P* < 0.001) (Fig 1C). An examination of the estimated marginal means revealed that males without neighbours are especially likely to honk during the breeding season (Fig 1C).

### Moans

We found that male giant pandas were significantly more likely to moan during the breeding season than outside of the reproductive period (Wald χ^2^_1, 992_ = 6.7, *P* = 0.034). In addition, neighbour context significantly affected the production of male moans (Wald χ^2^_2, 991_ = 4.9, *P* = 0.027) (Fig 1D). Males moaned more when they were housed next to other males than females (*P* = 0.049). No difference in moaning was revealed for males housed next to males versus no neighbours (*P* = 0.075). Although the interaction term season*social context was not significant and therefore not included in the model, it is notable that the breeding season increase in moaning is most apparent for males housed next to other males (Fig 1D).

### Barks and growls

Season (Wald χ^2^_1, 992_ = 0.5, *P* = 0.469) and neighbour context (Wald χ^2^_2, 991_ = 3.6, *P* = 0.166) did not significantly affect the production of male barks (Fig 1E). In addition, while there was a trend for males to produce more growls during the breeding season (Wald χ^2^_2, 991_ = 3.8, *P* = 0.051) (Fig 1E), neighbour context did not affect male growling behaviour (Wald χ^2^_2, 991_ = 0.0, *P* = 1.000). It is noteworthy that male giant pandas only produced growls when neighboured by females (Fig 1F).

## Discussion

The results of this study demonstrate that male giant pandas vary vocal signal production according to season and the sex of conspecific receivers. As predicted, males were significantly more likely to bleat when they were neighboured by female than male conspecifics during the breeding season. These results emphasise that bleats are crucial for signalling friendly intent and promoting contact between the sexes to facilitate reproduction. In addition, because only non-oestrous females served as neighbours in the current study, our findings suggest that bleats have an active role in initiating and controlling the interactions of a male and female prior to oestrus, and accord well with our previous contention that vocal familiarity might be important in female mate choice contexts in this species [45].

The reproductive strategy of giant pandas includes female advertisement to recruit multiple males, which then compete for access to the female [61]. It is conceivable then, that free-ranging females may become progressively familiarised to the vocalisations of high-quality males that can outcompete other rivals and maintain close contact to them. Since female giant pandas can discriminate between bleats from different males [45], future studies should use playback experiments to present females with male bleats; firstly to establish differing levels of familiarity for the bleats of different individuals in the lead up to oestrus, and then to determine whether females show spatial preferences for familiar versus unfamiliar bleats during their peak oestrous period.

Male giant pandas were also found to chirp more during the breeding season and, contrary to our predictions, males neighboured by females were significantly less likely to chirp than those without neighbours or neighboured by other males. The function of male chirps is not known [36, 39]. However, because we found that male chirps occurred predominantly during the breeding season but almost never when males had potential mates close by, our findings suggest these calls could be used by male giant pandas to attract the attention of females for mating purposes. In support of this contention, recent work suggests that chirps may be used by female giant pandas to elicit and maintain the attention of males that are reluctant to mate during close range inter-sexual encounters [37]. Interestingly, we also found that males with male neighbours chirped almost as much as those without neighbours, which could indicate that these calls remain important outside of reproductive contexts, potentially for signalling submissive intent in this non-gregarious species [39].

The production of male giant panda honks also differed according to season and social context. Males honked significantly more often during the breeding season, and as predicted, were most likely to honk in the no neighbour context at this time. These findings indicate that males could be using honks to gain the attention of potential mating partners during the breeding season. Giant panda honks are relatively short vocalisations with a fundamental frequency contour that rapidly drops to a lower frequency range at the start of the call [35, 36]. Vocal signals with rapid frequency sweeps are theorised to be easier to locate [62], and possibly more evocative [63]. Hence, male giant pandas could use honks to advertise location and stimulate potential mates to approach them in this species’ visually occluded bamboo forest environment. Because female giant pandas also honk [35, 36, 39] future studies should establish whether the acoustic structure of honks varies according to the sex and identity of the caller, and use playback experiments to determine whether giant pandas preferentially approach speakers broadcasting honks from opposite-sexed versus same-sexed conspecifics.

Male giant pandas also moaned significantly more often during the breeding season and altered the production of moans according to the sex of neighbouring conspecifics. Males produced more moans when they were housed next to other males than when they were housed next to potential mates (i.e. females) or had no neighbours. Because giant panda moans are generally assumed to indicate negative emotional states [36, 39], we suggest that male moaning in this context is likely to reflect aggression towards other rival males during the breeding season. Notwithstanding this, the prediction that male giant pandas would produce more of the highly aggressive call types (barks, growls and roars) when housed next to same-sexed individuals during the breeding season was not supported.

Male roaring was not observed and season and social context did not significantly affect the production of male barks or growls, however, the relatively rare occurrence of these calls precludes any firm conclusions about seasonal and contextual influences. The lack of roaring supports the assertion that these calls are only given during or just prior to fighting [39], which could not occur in the current study with the animals housed separately. Male growling when housed next to females outside of the breeding season could reflect the general intolerance that giant pandas have towards one another when they lack the motivation to reproduce. In addition, the observation that male giant pandas produced barks in the no neighbour context raises the possibility that these calls are not always used to signal aggressive intent during close-range interactions. Rather, our findings suggest that barks may also function to elicit attention, as they do in other mammals [64, 65].

Furthermore, because giant panda barks are graded vocalisations that range from tonal to harsh [35], it is possible that tonal versus harsh variants have different communicative functions. Domestic dogs (*Canis familiaris*) produce harsh barks when threatened, and more tonal barks to attract attention in isolation contexts [66]. Hence, the barks we observed in the no neighbour context during the breeding season could be more tonal variants that might be used to elicit attention. An analysis of the acoustic structure of barks produced in isolation contexts versus other social contexts during the breeding season, and playback of these bark variants to giant pandas, is certainly warranted.

Finally, as the giant panda is a species *vulnerable* to extinction [67], with conservation breeding programs largely in place to support conservation translocations, it is also important to consider that human caregiving/domestication can sometimes lead to vocalisations becoming generalised outside their original function due to relaxed selection pressures [68]. Any loss of original functionality would clearly be detrimental when animals are released back into the wild–a central goal of captive breeding efforts. We suggest that future studies document vocal signal usage in free-ranging giant pandas to determine whether our findings in captive animals generalise to wild populations. Recordings of wild giant panda vocalisations could be obtained using remote video and audio recording devices at known breeding sites, allowing vocal behaviour to be captured in different behavioural contexts. If required, conservation managers could then potentially tailor husbandry practices to minimise the chances that giant panda vocalisations lose their original function as an unwanted by-product of captive breeding efforts.

## Supporting information

S1 Audio Male bleat. (WAV)

S2 Audio Male chirp. (WAV)

S3 Audio Male moan. (WAV)

S4 Audio Male bark. (WAV)

S5 Audio Male growl. (WAV)

S6 Audio Male honk. (WAV)

S1 Dataset Excel spread sheet of the study data. (XLSX)
